# *N*-[4-(*N*-Cyclo­hexyl­sulfamo­yl)phen­yl]acetamide

**DOI:** 10.1107/S160053681000961X

**Published:** 2010-03-20

**Authors:** Islam Ullah Khan, Mehmet Akkurt, Faiza Anwar, Shahzad Sharif

**Affiliations:** a Materials Chemistry Laboratory, Department of Chemistry, Government College University, Lahore 54000, Pakistan; b Department of Physics, Faculty of Arts and Sciences, Erciyes University, 38039 Kayseri, Turkey

## Abstract

In the title compound, C_14_H_20_N_2_O_3_S, the cyclo­hexyl ring adopts a chair conformation: the four coplanar C atoms of this ring make a dihedral angle of 64.8 (2)° with the benzene ring. In the mol­ecule, an intra­molecular C—H⋯O contact generates an *S*(6) ring motif. In the crystal structure, mol­ecules are linked *via* inter­molecular N—H⋯O hydrogen bonds into two-dimensional layers propagating in (100).

## Related literature

For related structures, see: Sharif *et al.* (2010 ▶); Mariam *et al.* (2009*a* ▶,*b* ▶); Asiri *et al.* (2009 ▶); Khan *et al.* (2009 ▶); Arshad *et al.* (2008 ▶, 2009 ▶); Gowda *et al.* (2007*a* ▶,*b* ▶,*c* ▶); Haider *et al.* (2010 ▶). For bond-length data, see: Allen *et al.* (1987 ▶). For hydrogen-bond motifs, see: Bernstein *et al.* (1995 ▶). For puckering and asymmetry parameters, see: Cremer & Pople (1975 ▶); Nardelli (1983 ▶).
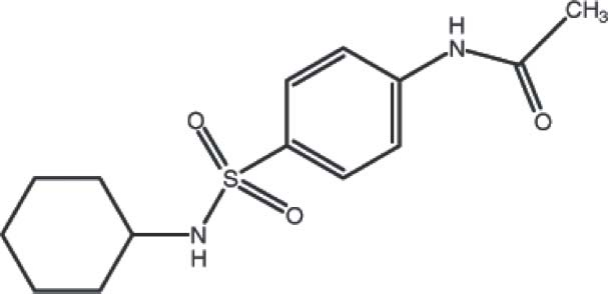


## Experimental

### 

#### Crystal data

C_14_H_20_N_2_O_3_S
*M* *_r_* = 296.39
Monoclinic, 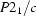

*a* = 14.6929 (19) Å
*b* = 13.3486 (19) Å
*c* = 7.9769 (12) Å
β = 102.387 (7)°
*V* = 1528.1 (4) Å^3^
*Z* = 4
Mo *K*α radiation
μ = 0.22 mm^−1^
*T* = 296 K
0.32 × 0.09 × 0.06 mm

#### Data collection

Bruker APEXII CCD diffractometer
11442 measured reflections
3628 independent reflections
1358 reflections with *I* > 2σ(*I*)
*R*_int_ = 0.110

#### Refinement

*R*[*F* ^2^ > 2σ(*F* ^2^)] = 0.062
*wR*(*F* ^2^) = 0.218
*S* = 0.94
3628 reflections
182 parameters
H-atom parameters constrained
Δρ_max_ = 0.30 e Å^−3^
Δρ_min_ = −0.38 e Å^−3^

### 

Data collection: *APEX2* (Bruker, 2007 ▶); cell refinement: *SAINT* (Bruker, 2007 ▶); data reduction: *SAINT*; program(s) used to solve structure: *SIR97* (Altomare *et al.*, 1999 ▶); program(s) used to refine structure: *SHELXL97* (Sheldrick, 2008 ▶); molecular graphics: *ORTEP-3 for Windows* (Farrugia, 1997 ▶); software used to prepare material for publication: *WinGX* (Farrugia, 1999 ▶) and *PLATON* (Spek, 2009 ▶).

## Supplementary Material

Crystal structure: contains datablocks global, I. DOI: 10.1107/S160053681000961X/hb5359sup1.cif

Structure factors: contains datablocks I. DOI: 10.1107/S160053681000961X/hb5359Isup2.hkl

Additional supplementary materials:  crystallographic information; 3D view; checkCIF report

## Figures and Tables

**Table 1:** Hydrogen-bond geometry (Å, °)

*D*—H⋯*A*	*D*—H	H⋯*A*	*D*⋯*A*	*D*—H⋯*A*
N1—H1⋯O3^i^	0.86	2.07	2.862 (4)	153
N2—H2⋯O2^ii^	0.86	2.11	2.970 (4)	177
C9—H9⋯O3	0.93	2.28	2.866 (5)	120

Symmetry codes: (i) 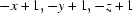; (ii) 
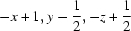
.

**Fig. 1.**
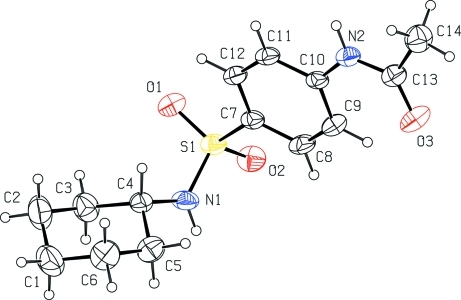
The molecule of (I) with displacement ellipsoids depicted at the 50% probability level for all non-H atoms.

**Fig. 2.**
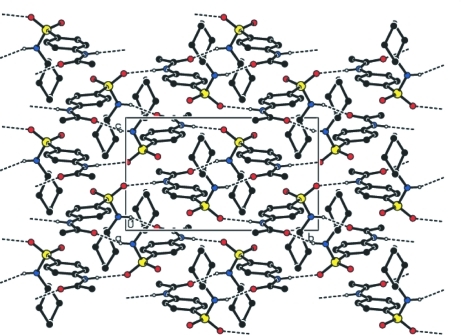
The packing and hydrogen bonding of (I) viewed down a-axis. Hydrogen bonding is indicated by dashed lines. For clarity, H atoms not involved in hydrogen bonding are omitted.

## supplementary crystallographic information

### Comment

The title compound (I), (Fig.1), was prepared and characterized as part of our ongoing studies of sulfonamide derivatives (Mariam *et al.*, 2009a,b; Sharif *et al.*, 2010).

The bond lengths (Allen *et al.*, 1987) and angles are within the normal ranges and are comparable to those in similar structures (Sharif *et al.*, 2010; Mariam *et al.*, 2009a,b; Asiri *et al.*, 2009; Khan *et al.*, 2009; Arshad *et al.*, 2008; Gowda *et al.*, 2007*a*,*b*,*c*; Haider *et al.*, 2010).

The C1–C6 cyclohexyl ring of (I) adopts a classic chair conformation [puckering parameters (Cremer & Pople, 1975) Q_T_ = 0.559 (6) Å, θ = 180.0 (6) ° and φ = 212 (16) °]. Atoms C1 and C4 deviate by -0.667 (6)Å and 0.639 (4) Å, respectively, from the plane through the other four atoms (C2,C3, C5 and C6) of the cyclohexane ring. The dihedral angle between the benzene ring (C7–C12) and the C2/C3/C5/C6 least-squares plane of the cyclohexane ring is 64.76 (20)° (Nardelli, 1983).

In the molecule of (I), intramolecular C—H···O hydrogen contacts generate *S*(5) and *S*(6) ring motifs (Bernstein *et al.*, 1995) (Table 1). In the crystal structure of (I), molecules are linked *via* intermolecular N—H···O hydrogen bonds into two-dimensional layers extended along the *b* axis (Table 1 and Fig. 2).

### Experimental

To 0.5 g ( 1.96 mmol ) *N*-acetyl *p*-amino sulfonyl chloride in 10 ml of distilled water was added 0.23 ml of cyclohexylamine (1.96 mmol) and stirring continued at room temperature, while maintaining the pH of the reaction mixture at 8 using 3% sodium carbonate. The progress of the reaction was continuously monitored by TLC. After consumption of all the reactants the mixture was filtered, dried and recrystalized from ethyl acetate to yield colourless needles of (I).

### Refinement

All H atoms were positioned geometrically and were treated as riding on their parent atoms, with N—H = 0.86 Å and C—H = 0.93-0.98 Å and *U*_iso_(H) = 1.2 or 1.5*U*_eq_(N, C).

### Crystal data

**Table:**

C_14_H_20_N_2_O_3_S	*F*(000) = 632
*M**_r_* = 296.39	*D*_x_ = 1.288 Mg m^−^^3^
Monoclinic, *P*2_1_/*c*	Mo *K*α radiation, λ = 0.71073 Å
Hall symbol: -P 2ybc	Cell parameters from 1027 reflections
*a* = 14.6929 (19) Å	θ = 3.0–18.7°
*b* = 13.3486 (19) Å	µ = 0.22 mm^−^^1^
*c* = 7.9769 (12) Å	*T* = 296 K
β = 102.387 (7)°	Needle, colourless
*V* = 1528.1 (4) Å^3^	0.32 × 0.09 × 0.06 mm
*Z* = 4	

### Data collection

**Table:**

Bruker APEXII CCD diffractometer	1358 reflections with *I* > 2σ(*I*)
Radiation source: sealed tube	*R*_int_ = 0.110
graphite	θ_max_ = 28.0°, θ_min_ = 1.4°
φ and ω scans	*h* = −19→16
11442 measured reflections	*k* = −17→16
3628 independent reflections	*l* = −10→9

### Refinement

**Table:**

Refinement on *F*^2^	Primary atom site location: structure-invariant direct methods
Least-squares matrix: full	Secondary atom site location: difference Fourier map
*R*[*F*^2^ > 2σ(*F*^2^)] = 0.062	Hydrogen site location: inferred from neighbouring sites
*wR*(*F*^2^) = 0.218	H-atom parameters constrained
*S* = 0.94	*w* = 1/[σ^2^(*F*_o_^2^) + (0.0926*P*)^2^] where *P* = (*F*_o_^2^ + 2*F*_c_^2^)/3
3628 reflections	(Δ/σ)_max_ < 0.001
182 parameters	Δρ_max_ = 0.30 e Å^−^^3^
0 restraints	Δρ_min_ = −0.38 e Å^−^^3^

### Special details

**Table:**

Geometry. Bond distances, angles etc. have been calculated using the rounded fractional coordinates. All su's are estimated from the variances of the (full) variance-covariance matrix. The cell esds are taken into account in the estimation of distances, angles and torsion angles
Refinement. Refinement on *F*^2^ for ALL reflections except those flagged by the user for potential systematic errors. Weighted *R*-factors wR and all goodnesses of fit *S* are based on *F*^2^, conventional *R*-factors *R* are based on *F*, with *F* set to zero for negative *F*^2^. The observed criterion of *F*^2^ > σ(*F*^2^) is used only for calculating -*R*-factor-obs etc. and is not relevant to the choice of reflections for refinement. *R*-factors based on *F*^2^ are statistically about twice as large as those based on *F*, and *R*-factors based on ALL data will be even larger.

### Fractional atomic coordinates and isotropic or equivalent isotropic displacement parameters (Å^2^)

**Table:**

	*x*	*y*	*z*	*U*_iso_*/*U*_eq_	
S1	0.68133 (8)	0.41022 (7)	0.21719 (15)	0.0554 (4)	
O1	0.7323 (2)	0.3332 (2)	0.1552 (4)	0.0645 (11)	
O2	0.6417 (2)	0.4904 (2)	0.1059 (4)	0.0679 (11)	
O3	0.2838 (2)	0.3369 (2)	0.5231 (4)	0.0801 (15)	
N1	0.7470 (2)	0.4618 (2)	0.3789 (5)	0.0619 (13)	
N2	0.3647 (2)	0.2116 (2)	0.4351 (4)	0.0515 (11)	
C1	0.9600 (4)	0.3914 (5)	0.8177 (8)	0.098 (3)	
C2	0.9654 (3)	0.3437 (5)	0.6484 (8)	0.101 (3)	
C3	0.9037 (3)	0.3989 (4)	0.4997 (7)	0.078 (2)	
C4	0.8045 (3)	0.4037 (3)	0.5194 (6)	0.0537 (16)	
C5	0.7987 (3)	0.4482 (4)	0.6899 (6)	0.0732 (19)	
C6	0.8608 (4)	0.3946 (4)	0.8377 (7)	0.085 (2)	
C7	0.5888 (3)	0.3520 (3)	0.2880 (5)	0.0468 (16)	
C8	0.5176 (3)	0.4092 (3)	0.3263 (6)	0.0585 (16)	
C9	0.4436 (3)	0.3657 (3)	0.3762 (6)	0.0553 (16)	
C10	0.4389 (3)	0.2626 (3)	0.3903 (5)	0.0437 (12)	
C11	0.5109 (3)	0.2061 (3)	0.3535 (5)	0.0488 (16)	
C12	0.5852 (3)	0.2493 (3)	0.3041 (5)	0.0483 (16)	
C13	0.2927 (3)	0.2488 (3)	0.4952 (5)	0.0533 (17)	
C14	0.2223 (3)	0.1742 (4)	0.5213 (7)	0.0773 (19)	
H1	0.74890	0.52610	0.38430	0.0750*	
H1A	0.99740	0.35330	0.91120	0.1180*	
H1B	0.98490	0.45900	0.82250	0.1180*	
H2	0.36500	0.14760	0.42250	0.0620*	
H2A	0.94580	0.27430	0.64800	0.1210*	
H2B	1.02940	0.34490	0.63450	0.1210*	
H3A	0.92720	0.46630	0.49340	0.0940*	
H3B	0.90600	0.36490	0.39320	0.0940*	
H4	0.77990	0.33530	0.51440	0.0650*	
H5A	0.73470	0.44490	0.70360	0.0870*	
H5B	0.81650	0.51820	0.69190	0.0870*	
H6A	0.85800	0.42850	0.94400	0.1010*	
H6B	0.83830	0.32670	0.84440	0.1010*	
H8	0.52010	0.47860	0.31800	0.0700*	
H9	0.39620	0.40550	0.40070	0.0660*	
H11	0.50880	0.13670	0.36270	0.0580*	
H12	0.63320	0.20960	0.28140	0.0580*	
H14A	0.17610	0.20640	0.57140	0.1160*	
H14B	0.25220	0.12230	0.59670	0.1160*	
H14C	0.19300	0.14550	0.41280	0.1160*	

### Atomic displacement parameters (Å^2^)

**Table:**

	*U*^11^	*U*^22^	*U*^33^	*U*^12^	*U*^13^	*U*^23^
S1	0.0707 (8)	0.0406 (6)	0.0528 (8)	−0.0034 (6)	0.0085 (6)	0.0005 (6)
O1	0.083 (2)	0.0502 (16)	0.065 (2)	0.0036 (15)	0.0264 (17)	−0.0055 (15)
O2	0.089 (2)	0.0530 (17)	0.060 (2)	−0.0026 (16)	0.0121 (17)	0.0160 (16)
O3	0.111 (3)	0.0498 (19)	0.088 (3)	0.0200 (18)	0.040 (2)	−0.0019 (18)
N1	0.077 (2)	0.0364 (18)	0.064 (3)	−0.0082 (17)	−0.003 (2)	−0.0040 (18)
N2	0.064 (2)	0.0383 (18)	0.051 (2)	−0.0023 (18)	0.0095 (18)	−0.0030 (16)
C1	0.078 (4)	0.114 (5)	0.089 (5)	−0.001 (3)	−0.013 (3)	0.001 (4)
C2	0.059 (3)	0.135 (5)	0.104 (6)	0.014 (3)	0.009 (3)	−0.004 (4)
C3	0.064 (3)	0.103 (4)	0.068 (4)	−0.003 (3)	0.017 (3)	−0.006 (3)
C4	0.056 (3)	0.040 (2)	0.062 (3)	−0.002 (2)	0.006 (2)	−0.002 (2)
C5	0.083 (3)	0.070 (3)	0.069 (4)	0.005 (3)	0.022 (3)	−0.003 (3)
C6	0.102 (4)	0.095 (4)	0.055 (4)	0.015 (3)	0.012 (3)	0.005 (3)
C7	0.062 (3)	0.040 (2)	0.033 (3)	0.001 (2)	−0.002 (2)	−0.0018 (18)
C8	0.079 (3)	0.030 (2)	0.065 (3)	0.000 (2)	0.012 (3)	−0.002 (2)
C9	0.071 (3)	0.038 (2)	0.060 (3)	0.006 (2)	0.021 (2)	0.001 (2)
C10	0.058 (2)	0.038 (2)	0.029 (2)	0.002 (2)	−0.0040 (19)	−0.0041 (18)
C11	0.068 (3)	0.031 (2)	0.043 (3)	0.000 (2)	0.002 (2)	−0.0018 (19)
C12	0.063 (3)	0.036 (2)	0.044 (3)	0.003 (2)	0.007 (2)	−0.0042 (18)
C13	0.071 (3)	0.051 (3)	0.036 (3)	0.007 (2)	0.007 (2)	0.001 (2)
C14	0.077 (3)	0.079 (3)	0.081 (4)	−0.001 (3)	0.028 (3)	−0.002 (3)

### Geometric parameters (Å, °)

**Table:**

S1—O1	1.422 (3)	C11—C12	1.365 (6)
S1—O2	1.432 (3)	C13—C14	1.482 (7)
S1—N1	1.592 (4)	C1—H1A	0.9700
S1—C7	1.761 (4)	C1—H1B	0.9700
O3—C13	1.209 (5)	C2—H2A	0.9700
N1—C4	1.471 (6)	C2—H2B	0.9700
N2—C10	1.395 (5)	C3—H3A	0.9700
N2—C13	1.347 (5)	C3—H3B	0.9700
N1—H1	0.8600	C4—H4	0.9800
N2—H2	0.8600	C5—H5A	0.9700
C1—C6	1.501 (9)	C5—H5B	0.9700
C1—C2	1.511 (9)	C6—H6A	0.9700
C2—C3	1.520 (8)	C6—H6B	0.9700
C3—C4	1.501 (6)	C8—H8	0.9300
C4—C5	1.503 (7)	C9—H9	0.9300
C5—C6	1.508 (7)	C11—H11	0.9300
C7—C12	1.379 (6)	C12—H12	0.9300
C7—C8	1.381 (6)	C14—H14A	0.9600
C8—C9	1.365 (6)	C14—H14B	0.9600
C9—C10	1.384 (6)	C14—H14C	0.9600
C10—C11	1.381 (6)		
			
O1—S1—O2	119.96 (19)	C1—C2—H2A	109.00
O1—S1—N1	108.76 (18)	C1—C2—H2B	109.00
O1—S1—C7	107.04 (18)	C3—C2—H2A	109.00
O2—S1—N1	105.91 (17)	C3—C2—H2B	109.00
O2—S1—C7	106.83 (19)	H2A—C2—H2B	108.00
N1—S1—C7	107.84 (19)	C2—C3—H3A	109.00
S1—N1—C4	122.6 (2)	C2—C3—H3B	109.00
C10—N2—C13	128.9 (3)	C4—C3—H3A	109.00
C4—N1—H1	119.00	C4—C3—H3B	109.00
S1—N1—H1	119.00	H3A—C3—H3B	108.00
C10—N2—H2	116.00	N1—C4—H4	108.00
C13—N2—H2	116.00	C3—C4—H4	108.00
C2—C1—C6	110.2 (5)	C5—C4—H4	108.00
C1—C2—C3	110.9 (5)	C4—C5—H5A	109.00
C2—C3—C4	111.7 (4)	C4—C5—H5B	109.00
N1—C4—C5	110.3 (4)	C6—C5—H5A	109.00
C3—C4—C5	110.9 (4)	C6—C5—H5B	109.00
N1—C4—C3	110.8 (4)	H5A—C5—H5B	108.00
C4—C5—C6	112.2 (4)	C1—C6—H6A	109.00
C1—C6—C5	111.7 (5)	C1—C6—H6B	109.00
C8—C7—C12	118.9 (4)	C5—C6—H6A	109.00
S1—C7—C8	120.0 (3)	C5—C6—H6B	109.00
S1—C7—C12	121.1 (3)	H6A—C6—H6B	108.00
C7—C8—C9	121.2 (4)	C7—C8—H8	119.00
C8—C9—C10	120.2 (4)	C9—C8—H8	119.00
N2—C10—C9	124.2 (4)	C8—C9—H9	120.00
N2—C10—C11	117.7 (3)	C10—C9—H9	120.00
C9—C10—C11	118.2 (4)	C10—C11—H11	119.00
C10—C11—C12	121.8 (4)	C12—C11—H11	119.00
C7—C12—C11	119.7 (4)	C7—C12—H12	120.00
O3—C13—C14	121.4 (4)	C11—C12—H12	120.00
N2—C13—C14	115.3 (4)	C13—C14—H14A	110.00
O3—C13—N2	123.3 (4)	C13—C14—H14B	109.00
C2—C1—H1A	110.00	C13—C14—H14C	109.00
C2—C1—H1B	110.00	H14A—C14—H14B	109.00
C6—C1—H1A	110.00	H14A—C14—H14C	109.00
C6—C1—H1B	110.00	H14B—C14—H14C	109.00
H1A—C1—H1B	108.00		
			
O1—S1—N1—C4	−46.6 (4)	C1—C2—C3—C4	−56.3 (6)
O2—S1—N1—C4	−176.8 (3)	C2—C3—C4—N1	177.1 (4)
C7—S1—N1—C4	69.2 (4)	C2—C3—C4—C5	54.4 (6)
O1—S1—C7—C8	−168.3 (3)	N1—C4—C5—C6	−176.8 (4)
O1—S1—C7—C12	10.9 (4)	C3—C4—C5—C6	−53.8 (5)
O2—S1—C7—C8	−38.6 (4)	C4—C5—C6—C1	55.2 (6)
O2—S1—C7—C12	140.5 (3)	S1—C7—C8—C9	177.9 (4)
N1—S1—C7—C8	74.8 (4)	C12—C7—C8—C9	−1.3 (7)
N1—S1—C7—C12	−106.0 (3)	S1—C7—C12—C11	−177.7 (3)
S1—N1—C4—C3	100.2 (4)	C8—C7—C12—C11	1.5 (6)
S1—N1—C4—C5	−136.7 (3)	C7—C8—C9—C10	0.4 (7)
C13—N2—C10—C9	−10.3 (6)	C8—C9—C10—N2	−177.9 (4)
C13—N2—C10—C11	171.5 (4)	C8—C9—C10—C11	0.3 (6)
C10—N2—C13—O3	−1.7 (7)	N2—C10—C11—C12	178.3 (4)
C10—N2—C13—C14	176.7 (4)	C9—C10—C11—C12	−0.1 (6)
C6—C1—C2—C3	56.5 (6)	C10—C11—C12—C7	−0.8 (6)
C2—C1—C6—C5	−56.0 (6)		

### Hydrogen-bond geometry (Å, °)

**Table:**

*D*—H···*A*	*D*—H	H···*A*	*D*···*A*	*D*—H···*A*
N1—H1···O3^i^	0.86	2.07	2.862 (4)	153
N2—H2···O2^ii^	0.86	2.11	2.970 (4)	177
C9—H9···O3	0.93	2.28	2.866 (5)	120

Symmetry codes: (i) −*x*+1, −*y*+1, −*z*+1; (ii) −*x*+1, *y*−1/2, −*z*+1/2.
